# Fetal programming effects of early weaning on subsequent parity calf performance

**DOI:** 10.1093/tas/txab049

**Published:** 2021-07-07

**Authors:** Jack L Oattes, Taoqi Shao, Parker A Henley, Daniel W Shike

**Affiliations:** Department of Animal Sciences, University of Illinois at Urbana-Champaign, Urbana, IL 61801, USA

**Keywords:** beef cow, early weaning, fetal programming

## Abstract

Mature Simmental × Angus cows [*n* = 147; body weight (BW) = 590 ± 72 kg] were used to evaluate the effects of early weaning on subsequent parity calf growth performance and carcass characteristics. Cows were assigned to one of two treatments based on their previous calf’s weaning age: early wean (EW) or conventional wean (CW). Dams assigned to the EW treatment had calves previously weaned at 88 ± 6 d of age, whereas dams assigned to the CW treatment had calves previously weaned at 185 ± 6 d of age. Cow BW and body condition scores (BCS) were monitored during the experiment. All cows were managed as a common group from the onset of the experiment at breeding until final pregnancy check of their next production cycle 462 d later. All calves in the experiment were managed as one group and weaned at a single time point, then feedlot performance and carcass characteristics were evaluated. Initial cow BW was different (*P* < 0.05), so it was included as a covariate for cow BW analysis. There was a treatment × date interaction (*P* < 0.01) for cow BW and cow BCS. Cow BW was consistently greater for the EW treatment from day 39 to the end of the experiment (*P* < 0.01). Cow BCS were not different at the onset of the experiment (*P* = 0.20), although after breeding and throughout lactation, BCS diverged between treatments and the EW treatment consistently had greater (*P* < 0.01) BCS than the CW treatment throughout the entire subsequent lactation. Gestation length was not different (*P* = 0.21) between treatments, yet calf birth BW was greater (*P* = 0.05) for the EW treatment. Neither artificially inseminated pregnancy percentage nor overall pregnancy percentage was different between treatments (*P* ≥ 0.61). Despite the greater birth BW for the EW treatment and no difference (*P* = 0.25) in milk production, weaning BW was not different (*P* = 0.50) between treatments. Feedlot performance measures were not different (*P* ≥ 0.13) between treatments, including feedlot arrival weight, final BW, average daily gain, dry matter intake, and gain to feed. Final carcass characteristics were not different (*P* ≥ 0.33) between treatments, including dressing percentage, hot carcass weight, LM area, 12th rib fat thickness, kidney, pelvic, heart fat percentage, USDA yield grade, and marbling score. Thus, early weaning resulted in improved BW and BCS of cows as well as increased birth BW of subsequent calf, although that did not transpire into differences in postnatal growth performance or carcass traits.

## INTRODUCTION

Cattle production requires producers to balance output with necessary inputs in a way that is both profitable and sustainable. Early weaning is a management tactic that can be employed in times of trying environmental conditions, which can lead to increased performance for the calf and aid in maintaining the cowherd in terms of body weight (BW), body condition score (BCS), and reproductive efficiency (Laster et al., 1973; Myers et al., 1999). If early weaning occurs, the cow is no longer required to commit nutrients to lactation and can divert more nutrients toward maintaining herself and potentially the growth of her fetus. Maternal nutrition is one of the major environment challenges that has persistent impacts on the performance of the progeny (Du et al., 2010). The idea that weaning age can impact lifelong performance and carcass characteristics by inducing a changed nutritional requirement of the dam during early gestation is a novel concept that has little to no research at this point. Some fetal programming work relative to early gestation has addressed an intrauterine growth restriction due to undernutrition and the resulting organogenesis, vascularization, and placental growth, as well as how that can ultimately affect postnatal growth and final carcass composition (Radunz et al., 2011; Vonnahme et al., 2003). The objective of this experiment was to determine the effects of early weaning and the accompanying change in cow nutrient requirements on the performance of the dam’s subsequent parity calf throughout the neonatal and postweaning feeding period as well as final carcass characteristics. It was hypothesized that cows lactating during early gestation will have greater nutrient requirements, which would lead to a potential negative energy balance, resulting in impaired fetal development leading to long-term effects on calf performance and carcass merit.

## MATERIALS AND METHODS

Experimental animals were managed according to the guidelines recommended in the Guide for the Care and Use of Agriculture Animals in Agriculture Research and Teaching (Federation of Animal Science Societies, 2010). All experimental procedures followed were approved by the University of Illinois Institutional Animal Care and Use Committee (IACUC Protocol #17151).

### Animals, Experimental Design, and Treatments

One hundred and eighty one fall-calving, mature, multiparous, Angus × Simmental cows (initial BW = 590 ± 72 kg, and initial BCS = 5.6 ± 0.78) were used to evaluate the effects of previous calf weaning age on subsequent parity calf growth performance and carcass characteristics. Cows were assigned to one of two treatments based on their previous calf’s weaning age: 1) early wean (EW) or 2) conventional wean (CW). The EW treatment had a steer calf weaned at 88 ± 6 d of age (day 0, December 15, 2016), whereas the CW treatment had a heifer calf that was weaned at 185 ± 6 d of age (day 97, March 22, 2017). No data are reported on these calves as that was not a part of the objectives of this experiment.

### Cow Management

Cows were synchronized using a 7-d CoSynch + CIDR protocol (Bremer et al., 2004) and were artificially inseminated (AI, day 0, December 15, 2016) to 10 Shorthorn bulls stratified across treatments. Only cows that had an AI-sired calf were included in the analysis. Cows were maintained as a single contemporary management group for the duration of the experiment. At the onset of the experiment (day 0), cows assigned to both treatment groups and calves from CW dams had ad libitum access to grass hay [acid detergent fiber (ADF) 44.1%, neutral detergent fiber (NDF) 75.1%, crude protein (CP) 7.8%] and were supplemented 1.8 kg dry matter (DM) of dried distillers grains/cow/d (DDGS; ADF 12.4%, NDF 38.7%, CP 30.3%, ether extract 12.3%). On day 40 (January 24, 2017), supplementation of DDGS was increased to 2.7 DM kg/cow/d until day 100 (March 25, 2017). On day 97, calves of CW cows were weaned.

On day 100, cows began rotationally grazing endophyte-infected tall fescue (*Festuca arundinacea*)/red clover (*Trifolium pratense*) mixed pastures (36.2% ADF, 64.4% NDF, and 9.8% CP). From day 344 to 371 (November 24, 2017 to December 21, 2017), cows continued to graze pastures and were supplemented 2.7 kg DM of DDGS (ADF 11.50%, NDF 43.85%, CP 24.57%, fat 11.51%). On day 372, cows were transitioned to 9.6 kg DM of a total mixed ration (TMR; 24% whole shelled corn, 12% soybean hulls, 32% DDGS, 28% ground hay, and 4% coproduct balancer on an as-fed basis; 66.5% NDF, 51% ADF, and 11.7% CP, on DM basis) until trial conclusion. Coproduct balancer in TMR contained 25.0% CP, 1.5% ether extract, 8.0% crude fiber, 14% Ca, 1% P, 0.5% Mg, 0.4% K, 3.5% NaCl, 300 mg/kg Cu, 3 mg/kg Se, 1500 mg/kg Zn, 24 kIU/kg Vitamin A, 2.4 kIU/kg Vitamin D3, and 25 IU/kg Vitamin E. For the duration of the experiment, cows had ad libitum access to salt and loose mineral (24.5% salt, 17.9% Ca, 9.4% Na, 3.0% P, 5.8% Mg, 0.06% K, 1500 mg/kg Cu, 26 mg/kg Se, 242.9 kIU/kg vitamin A, and 5.7 g/kg chlortetracycline). Subsequent calves for all cows were weaned on day 426 (February 14, 2018).

**Figure 1.**
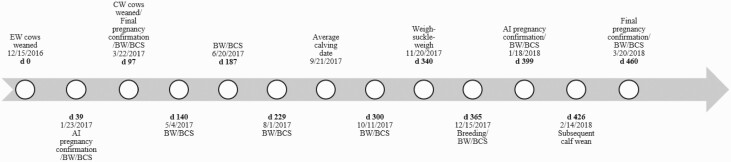
Timeline for cow management from initiation to final pregnancy confirmation during the subsequent parity.

Body weight and BCS on a 1–9 scale [1 = emaciated and 9 = obese, as described by Wagner et al. (1988)] were collected at 10 different time points from breeding on day 0 until postweaning on day 460 (March 20, 2018; Fig. 1). Milk production was estimated via the weigh-suckle-weigh technique (Boggs et al., 1980) at 60 ± 5.1 d postpartum (day 340) using a representative subset of cows from each treatment (*n* = 35 EW, *n* = 35 CW). Cows and calves were separated at 1200 h, allowed to nurse at 1900 h, and then were separated overnight. At 0700 h the next day, an empty calf BW was recorded, calves were allowed to nurse for 15 min, and a full calf BW was recorded. The BW difference between full and empty calf BW was estimated to be 12 h milk production. Estimate of 12 h milk production was multiplied by 2 to calculate 24 h milk production.

As per Dixon Springs Agriculture Center, cowherd’s annual vaccination schedule, cows received 2 mL Leptoferm 5 (Zoetis Inc., Parsippany, NJ) via intramuscular injection, and Ivermax pour on at 1 mL/10 kg BW topically. On day 187, cows received 1 mL anaplasmosis vaccine, 2 mL autogenous *Moraxella bovis*/*Moraxella bovoculi*, 2 mL Leptoferm 5 (Zoetis Inc., Parsippany, NJ), and 2 Corathon fly tags (Bayer, Pittsburgh, PA). On day 229, cows received 5 mL Bovishield Gold FP5VL5HB (Zoetis Inc. Parsippany, NJ), 2 mL Scourguard 4KC (Zoetis Inc., Parsippany, NJ), 5 mL Covexin 8 (Merck Animal Health, Madison, NJ), and 7 mL Mu-Se (Merck Animal Health, Madison, NJ). Cows were synchronized using a 7-d CoSynch + CIDR protocol (Bremer et al., 2004) and were AI (day 365, December 15, 2017) as a single group. After AI, all cows went to pasture and were exposed to five clean-up bulls from December 25, 2017 until February 15, 2018. Conception rates for AI pregnancy (34 d post-AI, January 17, 2018) and overall pregnancy (98 d post-AI, March 22, 2018) were determined via transrectal ultrasonography (Aloka 500, Hitachi Aloka Medical America, Inc., Wallingford, CT; 7.5 MHz general purpose transducer array).

### Calf Management

Upon birth, calf BW was taken within 24 h, calves received 1 mL vitamin AD, 1 mL Bo-Se (Merck Animal Health, Madison, NJ), 2 mL autogenous *Moraxella bovis*/*Moraxella bovoculi*, and 40 mL Bovisera (Colorado Serum Company, Denver, CO) all administered subcutaneously. All bull calves were castrated at birth. On day 399 (January 18, 2018), calves received 5 mL Bovishield Gold FP5VL5 HB (Zoetis Inc., Parsippany, NJ), 5 mL Covexin 8 (Merck Animal Health, Madison, NJ), 2 mL PulmoGuard PH-M (Agri-Labs, St. Joseph, MO), and 2 mL MpB Guard (Agri-Labs, St. Joseph, MO) all administered subcutaneously, as well as a 2-mL intranasal injection of Inforce 3 (Zoetis Inc. Parsippany, NJ). All calves in the experiment had one common weaning date, which was at 146 ± 5.1 d of age on day 426 (February 14, 2018). At weaning, calves received 5 mL Bovishield Gold FP5VL5 HB (Zoetis Inc. Parsippany, NJ), 5 mL Covexin 8 (Merck Animal Health, Madison, NJ), 2 mL PulmoGuard PH-M (Agri-Labs, St. Joseph, MO), and 2 mL MpB Guard (Agri-Labs, St. Joseph, MO) all administered subcutaneously. Additionally, from January 27, 2018 to February 1, 2018, calves received therapeutic oral dosage (5 mL) of Stress Care 5 AS 140 (Purina Animal Nutrition LLC, Gray Summit, MO), as well as Tetroxy 25 dispersible powder (Bimenda, US, Oakbrook Terrace, IL).

From the time of weaning (day 426), calves were maintained on pasture and fed an ad libitum postweaning diet consisting of 51.2% dry rolled corn, 34.5% dried distillers grains, 10% ground grass hay, and 4.3% of a Renaissance Nutrition Inc. (Roaring Springs, PA) coproduct balancer (25.0% crude protein, 1.5% crude fat, 8.0% crude fiber, 14% Ca, 1% P, 0.5% Mg, 0.4% K, 3.5% NaCl, 300 mg/kg Cu, 3 mg/kg Se, 1500 mg/kg Zn, 24 kIU/kg Vitamin A, 2.4 kIU/kg Vitamin D3, and 25 IU/kg Vitamin E) on a DM basis until being transported by commercial trucking on day 482 to the University of Illinois Beef and Sheep Research Facility in Urbana, IL, for the duration of the finishing period. Upon arrival to the University of Illinois Beef and Sheep Research Facility in Urbana, IL, calves were penned according to sex and incoming BW into five pens of heifers and five pens of steers with approximately 14 cattle per pen. Treatments were stratified with similar representation of treatment in each pen. Pens, 9.76 × 9.76 m in dimension, had concrete slatted floors covered by interlocking rubber matting and were constructed of 5.08-cm galvanized steel tubing. They were placed on a common receiving diet (Table 1) for 10 d following arrival, at which point they were transitioned to their common finishing diet using a single step-up diet (Table 1).

**Table 1.** Diet and nutrient composition of common feedlot diet

Item	Receiving diet^*b*^	Step-up diet^*c*^	Finishing diet^*d*^
Ingredient, % DM			
Dry rolled corn	30	30	30
MWDGS	25	25	20
Ground hay	10	–	–
High moisture corn	–	10	20
Corn silage	25	25	20
Supplement^*a*^	10	10	10
Analyzed values			
NDF, %	29.6	23.6	20.2
ADF, %	15.2	11.6	9.2
Fat, %	5.7	5.9	5.6
Protein, %	15.6	15.8	14.5

MWDGS, modified wet distillers grains.

^*a*^Supplement contained 73.4% ground corn, 17.8% limestone, 6.7% urea, 1.0% trace mineral premix, 0.17% Rumensin 90, 0.11% Tylan 40, and 0.84% fat. Trace mineral premix contained 8.5% Ca, 5% Mg, 7.6% K, 6.7% Cl, 10% S, 0.5% Cu, 2% Fe, 3% Mn, 3% Zn, 278 mg/kg Co, 250 mg/kg I, 150 mg/kg Se, 2,205 KIU/kg Vit A, 662.5 KIU/kg Vit D, 22,047.5 IU/kg Vit E. Tylan 40 (Elanco Animal Health, Greenfield, IN).

^*b*^Fed ad libitum from April 11, 2018 through April 21, 2018.

^*c*^Fed ad libitum from April 22, 2018 through May 1, 2018.

^*d*^Fed ad libitum from May 2, 2018 through slaughter.

Calves had BW collected every 28 d beginning on day 524 throughout the finishing period. Steers were implanted with Component TE-IS (16 mg estradiol and 80 mg trenbolone acetate; Elanco Animal Health) on day 524 and reimplanted with Component TE-S (24 mg estradiol and 120 mg trenbolone acetate; Elanco Animal Health) on day 608, whereas heifers were implanted with Component TE-IH (8 mg estradiol and 80 mg trenbolone acetate; Elanco Animal Health) on day 524 and reimplanted with Component TE-H (14 mg estradiol and 140 mg trenbolone acetate; Elanco Animal Health) on day 608. Individual feed intake was monitored using the GrowSafe automated feeding system (Model 4000E, GrowSafe Systems Ltd., Airdrie, Alberta, Canada) during the finishing period. Intakes were audited daily by trained personnel. Feed intake was considered acceptable if at least 85% of feed supplied to the bunk and 90% of corresponding feed assigned to individual ID was accounted for. Incidence of morbidity treatment during the finishing phase was recorded by animal care staff. Five calves received treatment for respiratory health during the finishing phase (EW = 3, CW = 2) and a total of four calves (EW = 3, CW = 1) were removed from the experiment as a result of mortality. Calves were fed 200 mg per calf per day of ractopamine hydrochloride (Optaflexx 45, 99 g/kg, Elanco Animal Health) for the last 30 d before slaughter. Calves were slaughtered at a commercial facility (Tyson Foods, Joslin, IL) on the same date, which was selected by visually appraising to target an average of 1.4-cm 12th rib fat thickness. Trained personnel recorded slaughter order and hot carcass weight (HCW) was taken on the day of slaughter. Carcass characteristics including 12th rib fat thickness, LM area, yield grade, and marbling score were taken after a 24-h carcass chill with Video Image Analysis as part of the USDA camera system. Carcass measurements from three steers from the EW treatment were excluded because of in-plant errors during data collection.

### Feed Sampling and Analysis

Samples of forage from pastures were hand clipped at the initiation of the pasture grazing period and monthly thereafter and composited for analysis. Dried distillers grain samples were collected monthly while being supplemented at the Dixon Springs Agricultural Center (DSAC) and composited for analysis. Hay and individual ingredient samples of TMR for cow diets were collected throughout the trial at DSAC and composited for analysis. Feed samples from DSAC were dried in a 55-°C forced air oven for 3 d and then ground with a Wiley mill (1-mm screen, Arthur H. Thomas, Philadelphia, PA). Individual ingredient feed samples from the common finishing diet fed at the University of Illinois Beef and Sheep Research Facility in Urbana, IL, were collected every 28 d over the course of the finishing phase, and individual ingredients were dried and ground, then composited at the conclusion of the experiment. All feed samples were analyzed for NDF and ADF (Goering and Van Soest, 1970; using Ankom Technology methods 5 and 6, respectively; Ankom200 Fiber Analyzer, Ankom Technology, Macedon, NY), fat (using Ankom Technology method 2; Ankom XT10 Fat Analyzer, Ankom Technology), CP (Leco TruMac, LECO Corporation, St. Joseph, MI), and ash (600 °C for 2 h; Thermolyne muffle oven Model F30420C, Thermo Scientific, Waltham, MA).

### Statistical Analysis

All data with the exclusion of binary data were analyzed as a completely randomized design using the MIXED procedures of SAS (SAS Inst. Inc., Cary, NC). Individual animal was experimental unit. All binary data were analyzed using the GLIMMIX procedure of SAS. For all repeated variables, unstructured covariance was selected using Akaike’s Information Criterion, as it provided the smallest Akaike information criterion. Date was added as a fixed effect for all repeated measures. Initial cow BW was included as a covariate for cow BW analysis. Cow age and days postpartum were used as covariates for the analysis of cow BW, BCS, and reproductive measures. Calf sex, sire, dam age, and pen were included in the model for calf parameters. Significance was declared at *P* ≤ 0.05. Means reported in tables are least squares means ± SEM.

## RESULTS AND DISCUSSION

**Figure 2.**
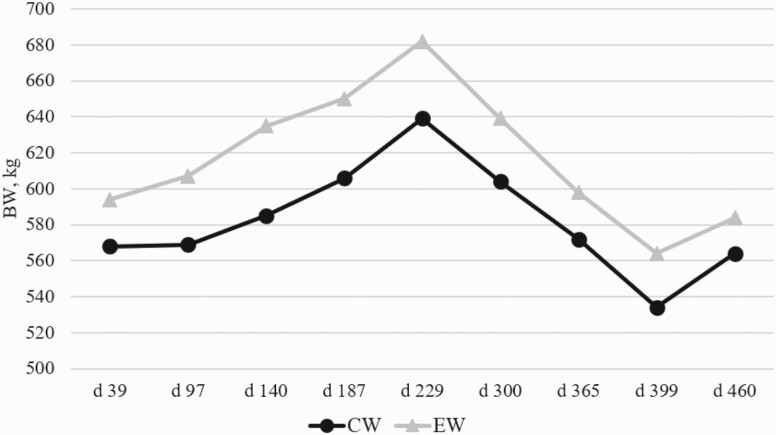
Influence of early weaning on cow BW. CW dams had previous calves weaned at 185 ± 6 d of age, and EW dams had previous calves weaned at 88 ± 6 d of age. BW was measured at EW wean date/breeding (day 0; included as covariate), AI pregnancy confirmation (day 39), CW date/final pregnancy confirmation (day 97), days 140, 187, and 229, postcalving evaluation (day 300), breeding (day 365), AI pregnancy confirmation (day 399), and final pregnancy confirmation (day 460). There was a treatment × date interaction (*P* < 0.01) for cow BW. On day 0, CON cows had greater (*P* < 0.05) BW than the EW cows. With BW on day 0 used as a covariate, cow BW was consistently greater for the EW treatment at all-time points throughout the experiment (*P* < 0.01).

**Figure 3.**
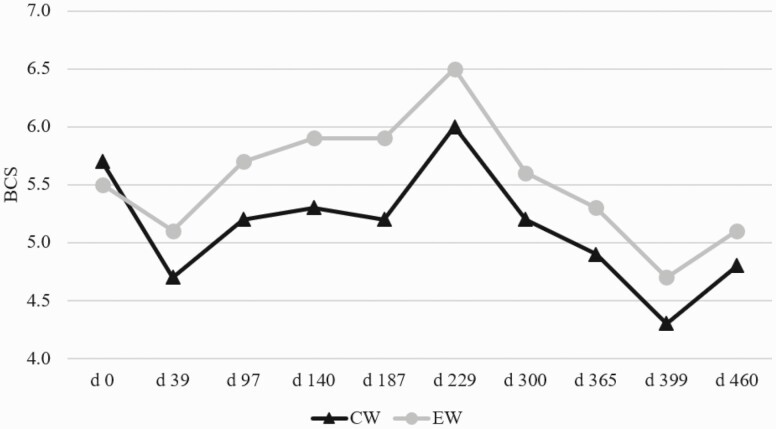
Influence of early weaning on cow BCS. CW dams had previous calves weaned at 185 ± 6 d of age, and EW dams had previous calves weaned at 88 ± 6 d of age. BCS was measured at EW date/breeding (day 0), AI pregnancy confirmation (day 39), CW date/final pregnancy confirmation (day 97), days 140, 187, and 229, postcalving evaluation (day 300), breeding (day 365), AI pregnancy confirmation (day 399), and final pregnancy confirmation (day 460). There was a treatment × date interaction (*P* < 0.01) for cow BCS. Cow BCS were not different at the onset of the experiment (*P* = 0.20), although after breeding and throughout lactation, BCS diverged between treatments and the EW treatment consistently had greater (*P* < 0.01) BCS than the CW treatment throughout the entire subsequent lactation.

Initial cow BW was different (*P* < 0.05), thus it was included as a covariate. There was a treatment × date interaction (*P* < 0.01) for cow BW and cow BCS. Cow BW was consistently greater for the EW treatment from day 39 to the end of the experiment (*P* < 0.01; Fig. 2). Cow BCS were not different at the onset of the experiment (*P* = 0.20; Fig. 3), although after breeding and throughout lactation, BCS diverged between treatments, and the EW treatment consistently had greater (*P* < 0.01) BCS than the CW treatment throughout the entire subsequent lactation. This is consistent with Arthington and Kalmbacher (2003) where cows that had an early weaned calf in January had achieved 39 kg more BW by the time of normal weaning in August and had a greater average BCS. Neville and McCormick (1981) discovered that dams of early weaned calves had 0.34 kg per day greater average daily gain (ADG) than cows with a normal weaned calf until the time of normal weaning.

Energy requirements were calculated (NASEM, 2016) to be 17.37 Mcal per day for the CW cows with 5.10 Mcal per day for lactation (120 d postparturition), 12.31 Mcal per day for maintenance, and 0.06 Mcal per day for gestation (day 45). The EW treatment had total energy requirements of 11.77 Mcal per day (NASEM, 2016) with 11.71 Mcal per day for maintenance and 0.06 Mcal per day for gestation (day 45). The CW cows lost both BW and BCS from the time of breeding on December 15, 2016 through January 23, 2017 when they would have reached peak lactation demands (NASEM, 2016). During that time, CW cows consumed 17.18 Mcal per day (1.8 kg of DDGS) and 19.16 Mcal (2.7 kg of DDGS) per day using NASEM (2016) energy values and assuming 1.75% of BW (10.17 kg per day) intake of grass hay. The CW cows had greater nutrition requirements during early gestation as they were still lactating. During the first 97 d of the trial, the CW cows lost BW and BCS indicating that they were deficient in nutrients for maintaining BW and BCS. Following weaning, CW cows regained BCS as would be expected, although at no point throughout another entire production cycle did CW cows match the BW or BCS of the EW treatment.

No observations tracking cows over another production cycle have been presented that would allow for comparison, although Neville and McCormick (1981) did track calf gestation length, which was longer for calves born from early weaned dams and weaning BW in subsequent progeny, which was not different. Cows from the EW treatment gained both BW and BCS postweaning, as was expected with the cessation of lactation and their NEm requirement at 11.771 Mcal per day for a mature cow during this period of gestation (NASEM, 2016). The decrease in BW and BCS of both treatments from August 1, 2017 until October 11, 2017 was associated with parturition. Milk production was not different (*P* = 0.25) between treatments, but no known literature in beef cattle has evaluated weaning age effects on subsequent lactation potential or carryover from length of a given lactation to the next. Both AI pregnancy percentage and overall pregnancy were not different between treatments in the year following weaning treatment (*P* ≥ 0.61; Table 2). No known literature has evaluated differences in pregnancy percentage the year following weaning treatment. It has been documented that weaning calves from cows before breeding season increases conception rates. Laster et al. (1973) conducted a trial where calves were weaned 8 d before the onset of a 42-d breeding period. Conception rates were greater for first and second parity cows whose calf had been weaned as compared to cows still lactating. First parity cows were 25.9% more likely to conceive in the 42-d breeding period, and second parity cows were 15.6% more likely to conceive. Also, Arthington and Kalmbacher (2003) reported early weaned cows had a 39.5% greater pregnancy rate during a 90-d breeding period that commenced in January at the time of early weaning. It is reported (Dziuk and Bellows, 1983; Houghton et al., 1990; Morrison et al., 1999) that BCS of 5 at calving could be considered a threshold for subsequent breeding success in mature beef cows. However, Richards et al. (1986) also reported that pregnancy rates of the cows with BCS equal or greater than 5 were improved by greater postpartum nutritional regimens. In the current study, the BCS of the EW cows was 5.3 and CW was 4.9 at time of subsequent AI. Although that is not a large difference in BCS between treatments, the CW cows were below the BCS threshold of 5. However, both treatment groups continued to lose BCS for the next 34 d. Further work with greater numbers is needed to fully evaluate the effect of early weaning on subsequent parity AI conception rate.

**Table 2.** Effects of previous calf weaning age on subsequent parity cow milk production and reproductive performance

	Treatment		SEM	*P*-value
Item	CW^*a*^	EW^*b*^		
Milk, kg^*c*^	7.1	6.4	0.41	0.25
AI pregnancy, %^*d*^	53	57	–	0.61
Overall pregnancy, %^*e*^	79	77	–	0.78

^*a*^Previous calf weaned at 185 ± 6 d of age.

^*b*^Previous calf weaned at 88 ± 6 d of age.

^*c*^Determined via weigh suckle weigh technique at 60 ± 5.1 d postpartum.

^*d*^Determined via transrectal ultrasonography at 34 d post-AI-breeding.

^*e*^Determined via transrectal ultrasonography at 98 d post-AI-breeding.

Gestation length was not different between treatments (*P* = 0.21; Table 3), while differences were observed in calf birth BW with EW cows having heavier calves (*P* = 0.05) than CW calves. The difference in birth BW did not carry over to weaning BW (*P* = 0.50) despite no milk yield difference (*P* = 0.25) detected. No previous work has been reported on early weaning fetal programming on subsequent parity calf performance. Given that CW cows experienced early gestation nutrient deficiency, studies that investigated nutrient restriction during gestation are discussed. In contrast from the current study, it was reported that cows fed 55% of NRC requirements during early gestation had calves with similar birth and weaning BW compared to the calves born from cows fed 100% NRC (Long et al., 2010). It was also reported that calves from dams fed hay (11.08 Mcal/d NEm and 840 g CP/d) during late gestation had lighter birth BW and did not compensate with postnatal growth and maintained lighter BW at the time of weaning compared to the calves from dams fed similar energy intakes but from corn or dried distillers grains with solubles (Radunz et al., 2012). Consistent with Radunz et al. (2012), in the current study, the cows (CW) with lower BCS during gestation had calves with lower birth BW. In contrast to the current study, Neville and McCormick (1981) reported calves in year 2 resulting from dams that had been part of the early wean treatment in year 1 calved 6–7 d later than their normal weaned counterparts and had greater 205-d weights than the progeny of dams that had weaned their calves normally in year 1. More studies on the effects of early weaning and different nutrient requirements on preweaning growth performance of the subsequent parity calf are needed to validate the effects observed in this experiment and test different strategies and scenarios.

**Table 3.** Effects of previous calf weaning age on subsequent parity calf performance during preweaning period

	Treatment			
Item	CW^*a*^	EW^*b*^	SEM	*P*-value
Birth BW, kg	37.6	39.4	0.82	0.05
Weaning BW, kg	152.9	155.6	4.12	0.50
Gestation length, days	279.6	280.6	0.76	0.21

^*a*^Dam’s previous calf weaned at 185 ± 6 d of age.

^*b*^Dam’s previous calf weaned at 88 ± 6 d of age.

Feedlot performance measures (Table 4) were not different between treatments including: feedlot arrival BW (*P* = 0.13); final BW (*P* = 0.66); ADG (*P* = 0.84); dry matter intake (DMI; *P* = 0.84); and gain to feed (G:F; *P* = 0.93). The results from studies that investigated the effects of maternal nutrition during gestation on finishing phase performance of the subsequent calves are mixed. Wilson et al. (2016) reported that greater prepartum dietary energy (125% total digestible nutrients [TDN] vs. 100% TDN requirement) during the last 83 d of gestation resulted in an increase in calf birth BW. Similar to the present experiment, that increase in calf birth BW did not translate into any further differences between treatments in performance throughout the finishing phase. On the other hand, Long et al. (2010) reported that steers from dams that had nutrient restriction during early gestation had greater initial finishing BW than the steers from dams fed nutritionally adequate diets and tended to have greater harvest weight. However, finishing-phase ADG of the steers in Long et al. (2010) was not influenced by prenatal treatment. Previous studies (Long et al., 2010; Wilson et al., 2016) as well as the current study indicate that maternal nutrient status, either during late or early gestation, could have limited effects on the growth performance of the offspring during finishing phase. The development of skeletal muscle, especially secondary myofibers, during early life has long-term effects on offspring growth and performance (Du et al., 2010). Skeletal muscle development in cattle initiates in embryonic stage, however, secondary myofibers form in the fetal stage between 2 mo and 7 or 8 mo of gestation (Du et al., 2010). In the current study, CW cows experienced nutrient deficiency during the first trimester of the gestation, which could also negatively impact the nutrient availability for the early embryo and lead to decreased birth BW but with limited effects on growth performance in finishing phase. It was reported that wether lambs from dams that were fed 50% NRC nutrient requirements during early to mid-gestation had greater live weight at 8 mo of age compared to the ones from dams fed 100% NRC requirements (Zhu et al., 2006). It is important to note that the 50% restriction was significantly greater than the present experiment. Given the mixed responses in the limited work that has been conducted, future work aimed at understanding underlying mechanisms for responses is needed.

**Table 4.** Effects of previous calf weaning age on subsequent parity calf performance post weaning

	Treatment			
Item	CW^*a*^	EW^*b*^	SEM	*P*-value
Feedlot arrival BW, kg	295	300	3.1	0.13
Final BW, kg	598	601	6.1	0.66
ADG, kg/d	1.61	1.61	0.028	0.84
DMI, kg/d	8.49	8.46	0.130	0.84
Gain to feed	0.19	0.19	0.002	0.93

^*a*^Dam’s previous calf weaned at 185 ± 6 d of age.

^*b*^Dam’s previous calf weaned at 88 ± 6 d of age.

The lack of difference between treatments continued postmortem with there being no differences between treatments in any measured carcass traits (Table 5), including dressing percentage (*P* = 0.33); HCW (*P* = 0.96); LM area (*P* = 0.94); 12th rib fat thickness (*P* = 0.73); kidney, pelvic, heart fat percentage (KPH; *P* = 0.80); USDA yield grade (*P* = 0.84); and marbling score (*P =* 0.70). Wilson et al. (2016) reported no differences in carcass parameters between late gestation dietary energy levels (125% TDN vs. 100% TDN requirement). In contrast, it was reported that ewes limit-fed DDGS during mid- and late-gestation had lambs with greater kidney and pelvic fat but lower dressing percentage and LM area compared to lambs from ewes fed haylage (Radunz et al., 2011). In addition, it was reported by Radunz et al. (2012) that calves from dams that were supplemented dried distillers grains and corn during mid- to late-gestation had lesser marbling scores than those which were fed isocaloric hay-based diet. Maternal nutrient restriction (50% NRC requirements) during early to mid-gestation decreased the number of myofibers and increased carcass weight and renal and pelvic fat in the lamb progeny (Zhu et al., 2006). Similarly, wethers from dams that had nutritional restriction (50% NRC requirement) from early to mid-gestation had greater BW and backfat than those from dams fed required nutrient level, mainly due to greater adiposity (Ford et al., 2007). Studies by the same group (Long et al., 2010; Long et al., 2012) indicated that nutrient restrictions during early to mid-gestation could affect body composition like reducing muscle mass. In the current study, nutrient deficiency for CW cows was not as severe, which led to limited effects on muscle development and carcass traits, like LM area. Marbling of beef is mainly determined by the density and sizes of intramuscular adipocytes. The formation of intramuscular fat mainly occurs from late fetal to about 250 d of age (Du et al., 2013), which provides sites for marbling formation during finishing phase. In the current study, the weaning treatments were applied to cows during early gestation of the subsequent parity calf, which might have limited effects on carcass characteristics.

**Table 5.** Effects of previous calf weaning age on subsequent parity calf carcass characteristics

	Treatment			
Item	CW^*a*^	EW^*b*^	SEM	*P*-value
Dressing %	61.6	61.3	0.002	0.33
HCW, kg	368	368	4.2	0.96
LM area, cm^2^	87.4	87.3	1.36	0.94
12th rib fat thickness, cm	1.45	1.47	0.050	0.73
KPH, %	2.0	2.0	0.03	0.80
USDA yield grade	3.2	3.2	0.11	0.84
Marbling score^*c*^	489	483	14.1	0.70

^*a*^Dam’s previous calf weaned at 185 ± 6 d of age.

^*b*^Dam’s previous calf weaned at 88 ± 6 d of age.

^*c*^400 = Choice USDA Quality Grade, 500 = Average Choice USDA Quality Grade.

## CONCLUSIONS

In conclusion, early weaning improved the BW and BCS of cows from the time of breeding throughout the next lactation. Birth BW of the subsequent parity calf was increased by EW. However, neither subsequent parity calf postnatal growth performance during preweaning and finishing period nor carcass characteristics was affected by the implementation of early weaning during gestation.
